# Left Ventricular Noncompaction Cardiomyopathy in an Elderly Patient: A Case Report

**DOI:** 10.7759/cureus.87020

**Published:** 2025-06-30

**Authors:** Reza Alavi, Hector Armando Sanchez Garcia, Mohamad Ahmad, Rami Akel

**Affiliations:** 1 Internal Medicine, Hospital Corporation of America (HCA) Florida Bayonet Point Hospital, Hudson, USA; 2 Cardiology, Hospital Corporation of America (HCA) Florida Bayonet Point Hospital, Hudson, USA; 3 Interventional Cardiology, Hospital Corporation of America (HCA) Florida Bayonet Point Hospital, Hudson, USA

**Keywords:** cardiomyopathy, echocardiography, left ventricular noncompaction cardiomyopathy, noncompacted-to-compacted ratio, structural heart disease, trabeculations

## Abstract

Left ventricular noncompaction is a rare cardiomyopathy, often underrecognized in older adults, characterized by prominent myocardial trabeculations and deep intertrabecular recesses. These structural abnormalities can lead to heart failure, arrhythmias, and thromboembolic events. We report the case of an 85-year-old woman with a history of heart failure with preserved ejection fraction and atrial fibrillation who presented with chest pain and dyspnea. Her electrocardiogram (ECG) revealed new diffuse T-wave inversions. Coronary angiography showed no obstructive disease. Transthoracic echocardiography demonstrated features consistent with left ventricular noncompaction cardiomyopathy (LVNC), including a noncompacted-to-compacted ratio greater than 2:1, along with a reduced left ventricular ejection fraction (LVEF) of 25-30% (normal LVEF >55%). Cardiac MRI was recommended to further characterize the myocardial structure, but the patient declined. She was managed with guideline-directed medical therapy for heart failure and anticoagulation and discharged with a wearable defibrillator. While LVNC is congenital, it may remain undiagnosed until later in life, particularly when symptoms arise due to progressive systolic dysfunction. This case illustrates the delayed diagnosis of a congenital condition rather than a late-onset disease. Greater awareness of LVNC and use of advanced imaging modalities, such as cardiac MRI, are important to improve recognition and management in older adults.

## Introduction

Left ventricular noncompaction cardiomyopathy (LVNC) is a rare and underrecognized form of cardiomyopathy resulting from incomplete myocardial compaction during embryogenesis. It is characterized by excessive trabeculations and deep intertrabecular recesses, which can lead to heart failure, arrhythmias, and thromboembolic events. The reported prevalence ranges from 0.01% to 0.3% in the general population, with recognition in elderly patients remaining uncommon, potentially due to underdiagnosis rather than true absence [1-3]. Early recognition is essential to prevent complications and guide therapy. Diagnosis is typically made using echocardiography, which includes criteria such as a two-layered myocardium with a noncompacted-to-compacted ratio greater than 2:1 in end-systole, multiple prominent trabeculations, and color Doppler evidence of deep recesses within the ventricular cavity [1-3]. Cardiac MRI can provide additional diagnostic confirmation, using Petersen’s criterion (noncompacted-to-compacted ratio >2.3 in diastole) or Jacquier’s criterion (>20% of trabeculated mass relative to global LV mass) [4-5]. Differentiating LVNC from mimickers, such as hypertrophic cardiomyopathy, apical thrombus, or normal prominent trabeculations, is crucial to avoid misdiagnosis. The clinical spectrum ranges from asymptomatic individuals to patients with severe systolic dysfunction or life-threatening arrhythmias. Genetic testing and family screening are advised, given associations with autosomal dominant, X-linked, and mitochondrial inheritance patterns [4-5]. Management strategies for LVNC are similar to those for other forms of heart failure, with the addition of anticoagulation therapy at a lower threshold due to the increased risk of thromboembolic events [6].

## Case presentation

An 85-year-old Caucasian woman with a history of heart failure with preserved ejection fraction (HFpEF), paroxysmal atrial fibrillation, hypertension, and type 2 diabetes mellitus presented to Bayonet Point Hospital with acute-onset substernal chest pain and exertional dyspnea. Her symptoms improved with sublingual nitroglycerin. On admission (hospital day 1), her high-sensitivity troponin was markedly elevated at 1393 ng/mL (normal <0.04 ng/mL). Differential diagnosis for this elevation included type 2 myocardial infarction due to supply-demand mismatch or myocarditis, particularly given the absence of obstructive coronary artery disease.

**Figure 1:**
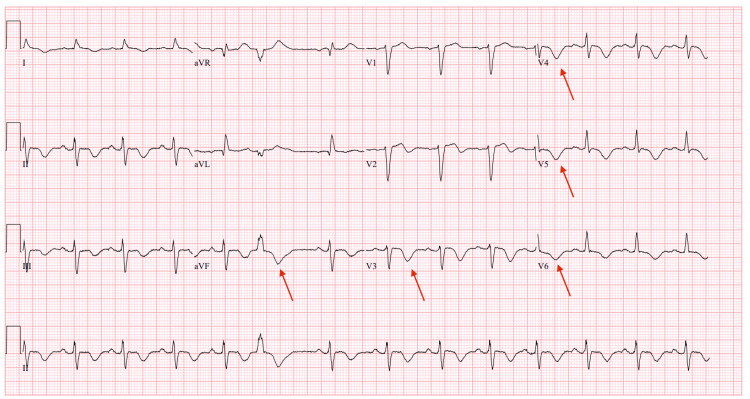
Admission ECG showing sinus rhythm with diffuse T-wave inversions The red arrows highlight these T-wave inversions.

**Figure 2:**
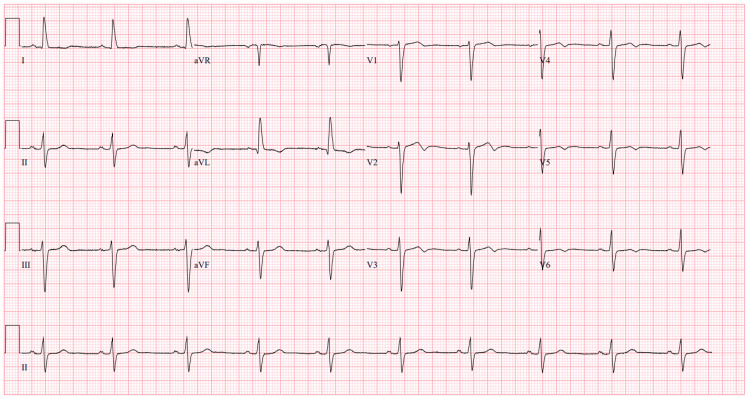
Baseline ECG prior to admission showed a normal sinus rhythm

**Figure 3:**
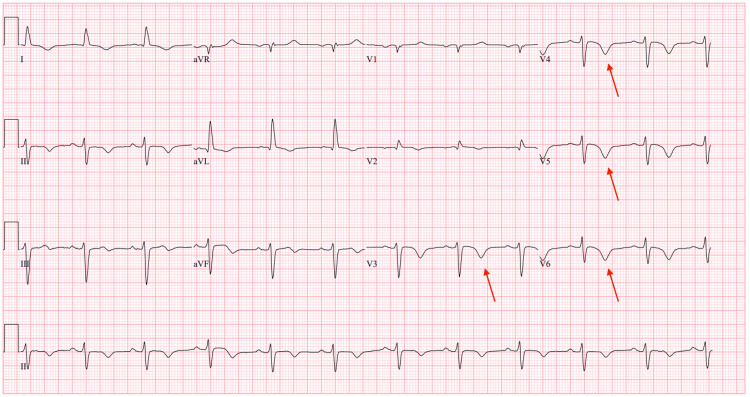
ECG before discharge showing partial resolution of T-wave changes The arrows show fewer T-wave inversions compared to the ECG shown in Figure 1.

An ECG on admission showed sinus rhythm with diffuse T-wave inversions (Figure 1), whereas her baseline outpatient ECG showed normal sinus rhythm without T-wave abnormalities (Figure 2). Repeat ECGs during hospitalization revealed progressive T-wave inversions, with partial resolution noted prior to discharge (Figure 3). This change could reflect resolving myocardial strain, treatment response, or nonspecific repolarization abnormalities of uncertain significance.

**Figure 4:**
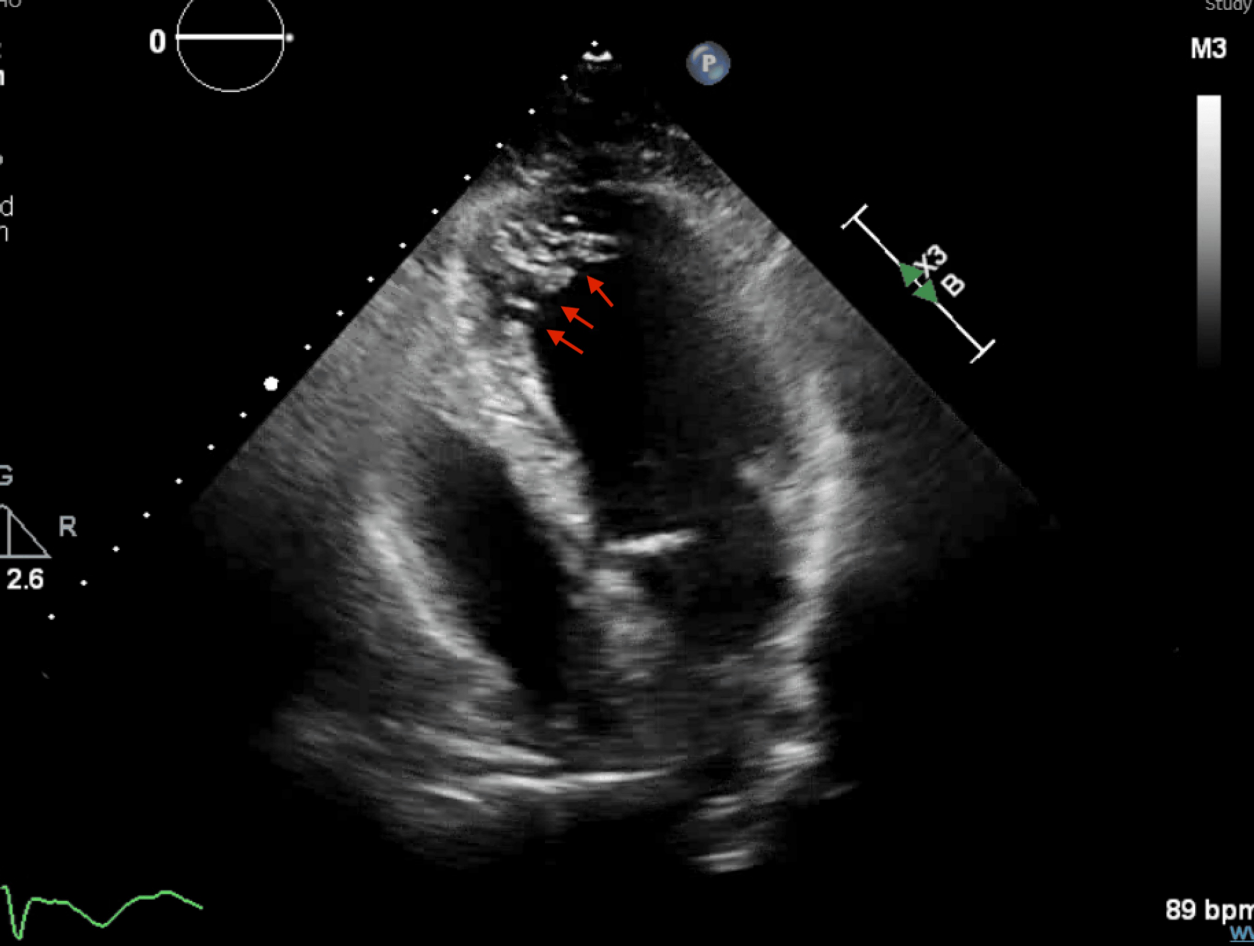
Echocardiographic image at end-systole demonstrating prominent trabeculations with a noncompacted-to-compacted myocardial ratio >2:1 The red arrows indicate some of the excessive trabeculations within the left ventricle. The noncompacted-to-compacted ratio was measured in end-systole using the Jenni criteria, confirming the diagnosis of left ventricular noncompaction.

**Figure 5:**
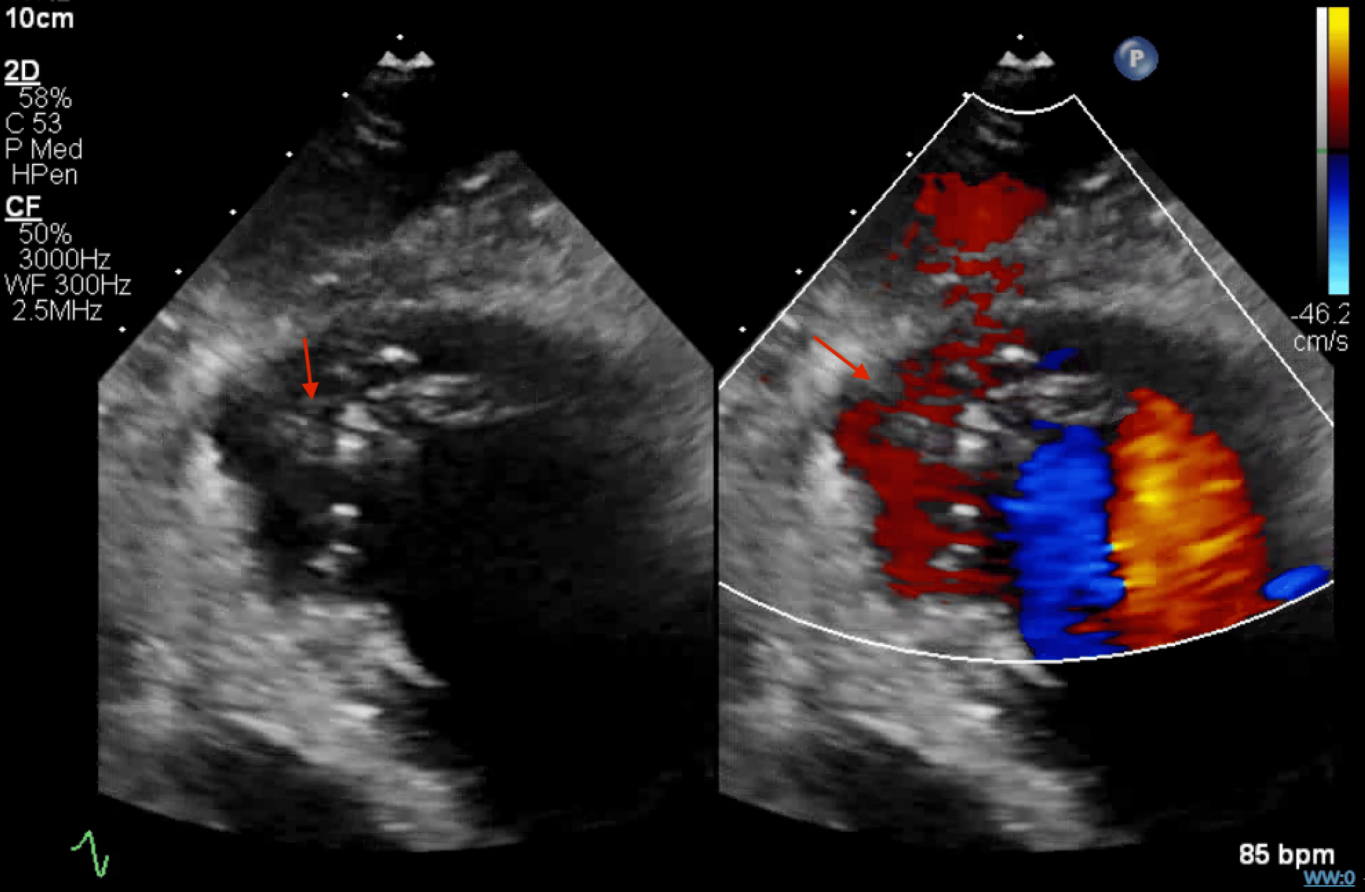
Color Doppler imaging in systole shows flow through intertrabecular recesses The red arrows show some of the trabeculations in the left ventricle.

Emergent coronary angiography (hospital day 1) revealed non-obstructive coronary arteries. Transthoracic echocardiography performed on hospital day 2 demonstrated a reduced left ventricular ejection fraction of 25-30% (normal >55%) and prominent trabeculations in the apical and mid-ventricular segments. Still frames captured at end-systole and end-diastole showed a noncompacted-to-compacted myocardial ratio >2:1 (Figure 4), meeting echocardiographic criteria for LVNC. Color Doppler imaging demonstrated flow within deep intertrabecular recesses (Figure 5), further supporting the diagnosis.

Cardiac MRI was offered to confirm the extent of noncompaction and assess for fibrosis, but the patient declined due to age and comorbidities, limiting definitive tissue characterization and volumetric assessment. Genetic testing and family screening were considered, given the congenital nature of LVNC and its autosomal dominant, X-linked, or mitochondrial inheritance patterns, but were deferred pending outpatient cardiology evaluation.

She was started on guideline-directed medical therapy, including metoprolol succinate, sacubitril-valsartan, dapagliflozin, and apixaban. She was discharged with a wearable cardioverter defibrillator and scheduled for outpatient cardiology follow-up to evaluate for implantable cardioverter-defibrillator placement.

## Discussion

This case underscores the importance of considering LVNC as a cause of new-onset systolic heart failure, particularly in elderly patients with overlapping comorbidities. LVNC can clinically or structurally mimic HFpEF or ischemic cardiomyopathy in older adults due to shared features such as diastolic dysfunction, reduced compliance, and symptoms of congestion despite preserved or mildly reduced ejection fraction [3,5]. Diagnostic pitfalls include attributing symptoms solely to hypertensive heart disease, atrial fibrillation, or coronary artery disease without evaluating for underlying structural cardiomyopathies [3].

The diagnosis of LVNC in this patient was notable for several reasons, including the atypical age of onset, the delay in diagnosis, the successful noninvasive diagnosis, and the presence of electrocardiographic abnormalities that served as early diagnostic clues. Although LVNC is more often recognized in pediatric populations, cases in older adults, while rare, have been reported, suggesting under-recognition rather than true absence [5,7].

One notable ECG finding in our patient was T-wave inversions in the inferolateral leads. These repolarization changes have been described in LVNC and may reflect subendocardial ischemia or architectural repolarization abnormalities due to excessive trabeculations. However, T-wave inversions are neither specific nor sensitive for LVNC and can be incidental or related to comorbid ischemic heart disease, limiting their diagnostic utility without supporting imaging [8-11].

The prognostic implications of LVNC in elderly patients remain uncertain, as most data derive from younger cohorts; however, potential risks include arrhythmias, thromboembolic events, and progressive heart failure, necessitating individualized risk stratification [3,5,6]. While device therapy, such as ICD implantation, is considered in LVNC patients with reduced ejection fraction, its utility in older adults requires assessment of the overall goals of care [3,5].

Genetic evaluation and family screening are standard in LVNC due to its hereditary nature, but in this case, testing was not pursued given the patient’s advanced age, absence of family history, and limited implications for her immediate management [4,5].

As highlighted in the literature, LVNC is often misdiagnosed or underrecognized in older adults, largely due to overlapping cardiac comorbidities and nonspecific presentations [3]. This case raises questions about the true prevalence of LVNC in the aging population and underscores the need for greater clinical vigilance to ensure appropriate diagnosis and management [3,5].

## Conclusions

This case illustrates the diagnostic challenges of LVNC in the elderly, particularly in patients with overlapping cardiovascular comorbidities. It underscores the importance of revisiting prior diagnoses and imaging when new cardiac dysfunction emerges. While echocardiography can often establish the diagnosis, advanced imaging modalities such as cardiac MRI may be essential in selected or equivocal cases to confirm the diagnosis and assess the extent of noncompaction. Early identification can significantly influence clinical management, as in this patient, where the diagnosis prompted guideline-directed medical therapy for heart failure and consideration of anticoagulation given the risk of thromboembolism. Although implantable defibrillator placement was not pursued due to the patient’s advanced age and goals of care, she was discharged with a wearable cardioverter-defibrillator for interim protection, as device therapy is often indicated in LVNC patients with reduced ejection fraction. Genetic evaluation and family screening were discussed but ultimately not pursued given her age and lack of immediate implications for her management. Future research should focus on defining the epidemiology of LVNC in older adults, validating diagnostic criteria in this population, and evaluating treatment outcomes to guide optimal care.
